# Risk factors associated with fatal pulmonary hemorrhage in locally advanced non-small cell lung cancer treated with chemoradiotherapy

**DOI:** 10.1186/1471-2407-12-27

**Published:** 2012-01-20

**Authors:** Masami Ito, Seiji Niho, Keiji Nihei, Kiyotaka Yoh, Hironobu Ohmatsu, Yuichiro Ohe

**Affiliations:** 1 Department of Thoracic Oncology, National Cancer Center Hospital East, Kashiwa, Chiba, Japan; 2 Department of Radiation Oncology, National Cancer Center Hospital East, Kashiwa, Chiba, Japan

## Abstract

**Background:**

The purpose of this study was to identify the risk factors associated with fatal pulmonary hemorrhage (PH) in patients with locally advanced non-small cell lung cancer (NSCLC), treated with chemoradiotherapy.

**Methods:**

The medical records of 583 patients with locally advanced NSCLC, who were treated with chemoradiotherapy between July 1992 and December 2009 were reviewed. Fatal PH was defined as PH leading to death within 24 h of its onset. Tumor cavitation size was defined by the cavitation diameter/tumor diameter ratio and was classified as minimum (< 0.25), minor (≥ 0.25, but < 0.5), and major (≥ 0.5).

**Results:**

Of the 583 patients, 2.1% suffered a fatal PH. The numbers of patients with minimum, minor, and major cavitations were 13, 11, and 14, respectively. Among the 38 patients with tumor cavitation, all 3 patients who developed fatal PH had major cavitations. On multivariate analysis, the presence of baseline major cavitation (odds ratio, 17.878), and a squamous cell histology (odds ratio, 5.491) proved to be independent significant risk factors for fatal PH. Interestingly, all patients with fatal PH and baseline major cavitation were found to have tumors with squamous cell histology, and the occurrence of fatal PH in patients having both risk factors was 33.3%.

**Conclusions:**

Patients at high risk of fatal PH could be identified using a combination of independent risk factors.

## Background

Clinical trials have shown that properly chosen candidates with locally advanced non-small cell lung cancer (NSCLC) have a survival advantage when treated with chemoradiotherapy, which is now a widely used mode of treatment for such patients [1,2].

Massive pulmonary hemorrhage (PH) is one of the most serious events observed in patients with lung cancer treated with chemotherapy and/or radiotherapy, and is now highlighted by the introduction of bevacizumab (Avastin; Genentech, South San Francisco, CA, USA), which induce a high incidence of massive PH in a subset of patients. Although several studies have evaluated risk factors that are suggested to be associated with the development of a massive PH in the setting of endobronchial brachytherapy or bevacizumab therapy [3-7], these reports included relatively small numbers of patients or had results that lacked sufficient statistical power. In addition, no previous reports have evaluated the risk factors of massive PH in the setting of chemoradiotherapy.

In the present report, we reviewed a large series of consecutive patients with locally advanced NSCLC treated with chemoradiotherapy. The purpose of this study was to identify risk factors associated with fatal PH in these patients.

## Methods

### Patients

A total of 598 patients with stage II and III NSCLC, treated with chemoradiotherapy between July 1992 and December 2009 were identified in our departmental database. Fifteen patients were excluded because a pre-therapy chest computed tomography (CT) scan was not available. The remaining 583 patients comprised the study cohort. Fatal PH, defined as a PH leading to death within 24 h of its onset, was determined by reviewing medical records. PH events that were possibly caused by an additional complicating factor such as disease progression were excluded.

### Radiographic tumor characteristics

Chest CT scans of all patients were assessed by a physician blinded to the clinical history and patient status. The presence and size of the cavitation as well as the longest diameter of the largest tumor mass were evaluated as potentially relevant baseline tumor characteristics. Cavitation size was defined as the cavitation diameter/tumor diameter ratio and was classified as minimum (< 0.25), minor (≥ 0.25 but < 0.5), or major (≥ 0.5).

### Clinicopathological information

We reviewed the regularly updated clinical database of each patient for the following clinicopathological information: age (below or above 70 years), gender, Eastern Cooperative Oncology Group performance status (0, 1, or 2), smoking history (nonsmokers or ever-smokers), TNM stage, tumor location (central or peripheral), tumor laterality (right or left), baseline chest pain (presence or absence), baseline cough (presence or absence), and baseline hemoptysis (presence or absence). We included age in the analysis using 70 years as a cut-off to provide the information on elderly patients, because 70 years is widely accepted as a cut-off point for defining the elderly population [8]. Disease stages was based on the TNM classification of the International Union Against Cancer, 6th edition [9]. We defined peripheral tumors as those in which the center of the mass was within the parenchyma and had no or minimal contact with hilar structures. Other tumors were labeled central tumors.

### Pathological evaluation

We reviewed the medical records of each patient for information on tumor pathology. Histological type was determined according to the World Health Organization classification [10].

### Statistical analysis

For univariate analyses, variables were evaluated using Fisher's exact test. For multivariate analysis, logistic regression was used to identify independent risk factors related to the incidence of fatal PH. All p values reported were 2-sided, and the significance level was set at less than 0.05. Analyses were performed using the statistical software SPSS 11.0 (Dr. SPSS II for Windows, standard version 11.0; SPSS Inc., Chicago, IL, USA). This study was conducted as part of a National Cancer Center institutional review board-approved protocol.

## Results

Table 1 shows the clinicopathological characteristics of 583 patients with locally advanced NSCLC. The patient cohort consisted of 482 (82.7%) men and 101 (17.3%) women. Their age range was 31-85 years with a median of 65 years. The most predominant histological type was adenocarcinoma (275; 47.2%), followed by squamous cell carcinoma (208; 35.7%). Of the 583 patients, 12 (2.1%) patients developed fatal PH. Centrally located tumors were seen in 127 (21.8%) patients. Baseline tumor cavitation was detected in 38 (6.5%) patients.

**Table 1:** Clinicopathological characteristics of 583 patients treated with chemoradiotherapy

Characteristics		No. of patients (%)
Overall number		583
Age (years)	median (range)	65 (31-85)
	≤ 70	449 (77.0)
	> 70	134 (23.0)
Gender		
	Male	482 (82.7)
	Female	101 (17.3)
ECOG PS		
	0	171 (29.3)
	1	408 (70.0)
	2	4 (0.7)
Smoking history		
	Nonsmoker	51 (8.7)
	Ever-smoker	532 (91.3)
Stage		
	IIA	6 (1.0)
	IIB	24 (4.1)
	IIIA	209 (35.8)
	IIIB	344 (59.0)
Histology		
	Adenocarcinoma	275 (47.2)
	Squamous cell carcinoma	208 (35.7)
	NSCLC	98 (16.8)
Fatal pulmonary hemorrhage		
	Absent	571 (97.9)
	Present	12 (2.1)
Tumor loction		
	Central	127 (21.8)
	Peripheral	456 (78.2)
Tumor laterality		
	Right	346 (59.3)
	Left	236 (40.5)
Baseline tumor cavitation		
	Absent	545 (93.5)
	Present	38 (6.5)
Baseline cough		
	Absent	317 (54.4)
	Present	266 (45.6)
Baseline chest pain		
	Absent	484 (83.0)
	Present	99 (17.0)
Baseline sputum		
	Absent	454 (77.9)
	Present	129 (22.1)
Baseline hoarseness		
	Absent	536 (91.9)
	Present	47 (8.1)
Baseline hemoptysis		
	Absent	468 (80.3)
	Present	115 (19.7)

ECOG PS: Eastern Cooperative Oncology Group Performance Status, NSCLC: non-small cell lung cancer

Table 2 shows the incidence of fatal PH and cavitation diameter/tumor diameter ratio in 38 patients with baseline tumor cavitation. The number of patients with minimum, minor, and major cavitations were 14, 11, and 13, respectively. Among the patients with cavitation, all 3 patients with fatal PH had major cavitations.

**Table 2:** Incidence of fatal PH by baseline tumor cavitation diameter/tumor diameter ratio

	No. of patients (n = 38)	
	
Cavitation diameter/tumor diameter	Non-fatal PH	Fatal PH
≥ 0.5	11	3
≥ 0.25, but < 0.5	11	0
< 0.25	13	0

PH: pulmonary hemorrhage

On univariate analysis, squamous cell histology, a centrally located tumor, and the presence of a major cavitation proved to be significant risk factors for fatal PH (Table 3). On multivariate analysis, squamous histology (odds ratio [OR], 5.491; *p *= 0.040; 95% confidence interval [CI], 1.079-27.943) and the presence of baseline tumor cavitation (OR, 17.878; *p *= 0.001; 95% CI, 3.430-93.190) were shown to be independent significant risk factors for fatal PH (Table 4).

**Table 3:** Correlation between fatal PH and pathological characteristics

		No. of patients (%)		*P *value†
			
Characteristics		Non-fatal PH	Fatal PH	
Overall number		571	12 (2.1)	
Age (y)	median (range)	65 (31-85)		
	≤ 70	437 (76.5)	12 (100)	0.078
	> 70	134 (23.5)	0 (0)	
Gender				
	Male	472 (82.7)	10 (83.3)	1.000
	Female	99 (17.3)	2 (16.7)	
ECOG PS				
	0	170 (29.8)	1 (8.3)	0.196
	1, 2	401 (70.2)	11 (91.7)	
Smoking history				
	Non smoker	51 (8.9)	0 (0)	0.613
	Ever-smoker	520 (91.1)	12 (100)	
Histology				
	Squamous cell carcinoma	198 (34.7)	10 (83.3)	< 0.001*
	Nonsquamous NSCLC	373 (65.3)	2 (16.7)	
TNM stage				
	II	30 (5.3)	0 (0)	1.000
	III	541 (94.7)	12 (100)	
Tumor laterality				
	Right	341 (59.7)	5 (41.7)	0.242
	Left	230 (40.3)	7 (58.3)	
Tumor location				
	Central	121 (21.2)	6 (50.0)	0.028*
	Peripheral	450 (78.8)	6 (50.0)	
Major cavitation				
	Absent	560 (98.1)	9 (75.0)	< 0.001*
	Present	11 (1.9)	3 (25.0)	
Baseline cough				
	Absent	313 (54.8)	4 (33.3)	0.155
	Present	258 (45.2)	8 (66.7)	
Baseline chest pain				
	Absent	475 (83.2)	9 (75.0)	0.438
	Present	96 (16.8)	3 (25.0)	
Baseline hemoptysis				
	Absent	458 (80.2)	10 (83.3)	1.000
	Present	113 (19.8)	2 (16.7)	

* indicates significance, †Fisher's exact test, PH: pulmonary hemorrhage, ECOG PS: Eastern Cooperative Oncology Group Performance Status, NSCLC: non-small cell lung cancer, Major cavitation is defined as its cavitation/tumor diameter ratio ≥ 0.5

**Table 4:** Multivariate analysis of risk factors for fatal pulmonary hemorrhage

		No. of patients (%)	Multivariate analysis		
Characteristics			OR	95% CI	*P*-value
Total		583 (100)			
Tumor location					
	Peripheral	456 (78.2)	1		
	Central	127 (21.8)	3.003	0.771-11.695	0.113
Histologic type					
	Nonsquamous NSCLC	375 (64.3)	1		
	Squamous cell carcinoma	208 (35.7)	5.491	1.079-27.943	0.040*
Major cavitation					
	Absent	569 (97.6)	1		
	Present	14 (2.4)	17.878	3.430-93.190	0.001*

OR: odds ratio, CI: confidence interval, * indicates significance, NSCLC: non-small cell lung cancer

The association between tumor histological type and the incidence of fatal PH among the patients with baseline major cavitation (n = 14) is shown in Table 5. Among the 14 patients with baseline major cavitation, fatal PH occurred in 3, all of whom had squamous cell carcinomas. The incidence of fatal PH in patients with both baseline major cavitation and squamous cell histology was 3/9 (33.3%).

**Table 5:** Association between tumor histology and incidence of fatal PH among patients with baseline major cavitation (n = 14)

	No. of patients (%)	
	
Histology	Non-fatal PH	Fatal PH
Overall number	11	3
Adenocarcinoma	5 (100)	0 (0)
Squamous cell carcinoma	6 (66.7)	3 (33.3)

## Discussion

The treatment of locally advanced NSCLC remains controversial [11] due to the heterogeneity of this patient population. Surgery alone is not recommended as the standard therapy, because the prognosis of these patients varies according to the status of mediastinal lymph node involvement. Additionally, primary surgery has been shown to have poor outcomes in certain subgroups of patients with this disease [12]. The most common treatment approaches are concurrent chemoradiation (CRT) [13,14], or in some cases trimodality therapy, which involves CRT followed by surgical resection [15,16]. Thoracic irradiation may cause a bronchovascular fistula, which results either from the rapid regression of the tumor or from necrosis of the bronchial mucosa and the vessel wall by radiotherapy itself with the attendant endothelial damage causing vascular abnormalities. As the pathogenesis of PH involves bronchovascular fistulae and vascular abnormalities [5,17], thoracic irradiation is a potential cause of PH, and its toxicity may be enhanced when combined with chemotherapy [18].

This study was conducted to identify the risk factors for fatal PH in patients with locally advanced NSCLC treated with chemoradiotherapy. Risk factors for fatal PH in this set of patients have not yet been established. In the current study, risk factors were determined by multiple logistic regression of variables that included patient demographics, baseline hemoptysis, tumor location, histological type, and baseline tumor cavitation, all of which could influence the occurrence of PH. We identified 2 independent significant risk factors for fatal PH: the presence of baseline major cavitation and squamous cell histology. The presence of baseline major cavitation proved to be a powerful risk factor (OR, 17.878) for fatal PH. Chaudhuri et al. examined cavitating lung cancer and reported that vascular invasion by tumor cells causes intratumoral ischemia [19], which induces hypoxia-inducible transcription factors and several angiogenic factors such as vascular endothelial growth factors [20]. The mechanism of PH in tumors with cavitation remains unclear, but disruption of the abnormal intratumoral vasculature by these transcription factors or cytokines may be one of the causes of PH in case of tumors with cavitation.

While the incidence of PH appeared to be high in patients with squamous cell carcinoma, it remains unclear whether squamous cell carcinoma contributed directly to the hemorrhage. For example, the risk of PH observed in the first phase I clinical trial of bevacizumab rendered antiangiogenic therapies inaccessible to patients with squamous cell carcinoma; [21] nevertheless, bevacizumab was added to standard frontline chemotherapy for NSCLC and has shown a survival benefit in these patients compared to chemotherapy alone [22,23]. However, it was not clear whether histology alone was the central risk factor for PH since squamous cell tumors differed from adenocarcinomas in that they were more frequently centrally located and had a greater tendency to cavitate. In the current study, we showed that squamous cell histology was associated with PH, independent of tumor location or the presence of cavitation.

Interestingly, the tumors of all patients with fatal PH who had major tumor cavitation before treatment had squamous cell histology. The incidence of fatal PH in patients having both risk factors, baseline major cavitation and squamous cell histology, was 33.3%; in contrast, the overall incidence in the study cohort was 2.1%. This findings shows that use of a combination of independent significant risk factors may enable the identification of patients at high risk of fatal PH.

Considering the risk factors for fatal PH identified here, primary surgery may be one of the treatment options for patients with operable locally advanced NSCLC, with baseline major cavitation and squamous cell histology. For the management of massive and recurrent hemoptysis, bronchial artery embolization (BAE) is also a demonstrated treatment option [24-31]; however, the bleeding recurrence rate among patients with BAE-treated lung cancer can reach 50% [32]. Surgical intervention, in contrast, is curative [33] and an established treatment for PH. A surgical approach may be beneficial for these patients at high risk of fatal PH with regard to local control of the potential source of the hemorrhage. In addition, although it remains unclear whether chemoradiation contributes directly to the occurrence of fatal PH in this study, primary surgical resection may help avoid potentially unfavorable primary chemoradiation.

The present study was retrospective and had limitations. It was not designed to evaluate the association between therapeutic modality and fatal PH risk. To our knowledge, no definitive information evaluates the direct association between therapeutic modality and fatal PH risk. Accordingly, it remains unclear which therapeutic modality among surgery, chemotherapy, and radiotherapy should be selected for patients identified as being at high risk of fatal PH in our study. We consider primary surgery to be a potential treatment option for patients with operable locally advanced NSCLC, who have both baseline major cavitation and squamous cell histology. However, further studies are required to compare the risk of surgery and the risk of fatal PH that accompanies chemotherapy or radiotherapy. Additionally, a considerable number of patients with locally advanced NSCLC are inoperable. For these patients, further studies are required in order to compare the differences between therapeutic modalities with regard to treatment benefit and the risk of fatal PH accompanying each therapy. Furthermore, since patients with fatal PH were all ≤ 70 years of age or had stage III tumors, we could not evaluate the correlation between age or stage and risk of fatal PH in this study. Despite these limitations, this is the first study to identify the statistically significant independent risk factors for fatal PH in patients with locally advanced NSCLC treated with chemoradiotherapy. We believe that our data will be helpful for future trials and for clinicians when determining therapeutic strategies for patients with locally advanced NSCLC.

## Conclusions

PH is a rare but life-threatening event that occurs in NSCLC. Patients at high risk of fatal PH may be identified by a combination of the independent risk factors--major baseline cavitation and squamous cell histology.

## Abbreviations

NSCLC: Non-small cell lung cancer; PH: Pulmonary hemorrhage; CT: Computed tomography; CI: Confidence interval; CRT: Chemoradiation; BAE: Bronchial artery embolization.

## Competing interests

The authors declare that they have no competing interests.

## Authors' contributions

MI contributed to the design and coordination of the study, performed the statistical analysis, prepared the manuscript, and read and approved the final manuscript. SN contributed to the design and coordination of the study, revised the article for important intellectual content, and read and approved the final manuscript. KH, KY, HO, and YO contributed to preparing the manuscript, and read and approved the final manuscript.

## Pre-publication history

The pre-publication history for this paper can be accessed here:

http://www.biomedcentral.com/1471-2407/12/27/prepub
